# Red algal Rubisco fails to accumulate in transplastomic tobacco expressing *Griffithsia monilis RbcL* and *RbcS* genes

**DOI:** 10.1002/pld3.45

**Published:** 2018-02-28

**Authors:** Myat T. Lin, Maureen R. Hanson

**Affiliations:** ^1^ Department of Molecular Biology and Genetics Cornell University Ithaca NY USA

**Keywords:** chloroplast transformation, *Griffithsia monilis*, photosynthesis, Rubisco

## Abstract

In C_3_ plants, the carbon fixation step catalyzed by ribulose‐1,5‐bisphosphate carboxylase/oxygenase (Rubisco) represents a major rate‐limiting step due to the competing oxygenation reaction, which leads to the energy‐intensive photorespiration and lowers the overall photosynthetic efficiency. Hence, there is great biotechnological interest in replacing the Rubisco in C_3_ crops with a more efficient enzyme. The Rubisco enzymes from red algae are among the most attractive choices due to their remarkable preference for carboxylation over oxygenation reaction. However, the biogenesis of Rubisco is extremely complex. The Rubisco enzymes in plants, algae, and cyanobacteria are made up of eight large and eight small subunits. The folding of the large subunits and the assembly of the large subunits with the small subunits to form a functional holoenzyme require specific chaperonin complexes and assembly factors. As a result, previous success in expressing foreign Rubisco in plants has been limited to Rubisco large subunits from closely related plant species and simpler bacterial enzymes. In our previous work, we successfully replaced the Rubisco in tobacco with a cyanobacterial enzyme, which was able to support the phototrophic growth of the transgenic plants. In this work, we used the same approach to express the Rubisco subunits from the red alga *Griffithsia monilis* in tobacco chloroplasts in the absence of the tobacco Rubisco large subunit. Although the red algal Rubisco genes are being transcribed in tobacco chloroplasts, the transgenic plants lack functional Rubisco and can only grow in a medium containing sucrose. Our results suggest that co‐expression of compatible chaperones will be necessary for successful assembly of red algal Rubisco in plants.

## 1 INTRODUCTION

A promising concept to increase crop yields is to improve plant productivity through more efficient photosynthesis (Long, Zhu, Naidu, & Ort, 2006; Ort et al., 2015). Several steps in the photosynthetic process in plants have been identified as potential targets for improvement, and some of them have already been implemented in model plants with notable success (Kromdijk et al., 2016; Simkin et al., 2017; Zhu, de Sturler, & Long, 2007). One major limitation in photosynthesis that has been challenging to overcome is the carbon fixation step, which is catalyzed by a highly inefficient enzyme known as ribulose‐1,5‐bisphosphate carboxylase/oxygenase (Rubisco) (Whitney, Houtz, & Alonso, 2011).

Rubisco carries out two competing reactions: carboxylation and oxygenation of ribulose‐1,5‐bisphosphate (RuBP) with the latter leading to photorespiration and lowering the overall photosynthetic efficiency. The CO_2_/O_2_ specificity factor (*S*_C/O_) of Rubisco is defined as the ratio of its carboxylation efficiency to its oxygenation efficiency. Generally, Rubisco with a higher *S*_C/O_ would be expected to provide higher carbon fixation efficiency. However, the evolutionary pathway of Rubisco has optimized its catalytic ability in a narrow kinetic landscape such that the enzyme with a high *S*_C/O_ has a low catalytic rate (*k* ^c^_cat_) and *vice versa* (Savir, Noor, Milo, & Tlusty, 2010; Tcherkez, Farquhar, & Andrews, 2006). For example, the Rubisco enzymes from C_3_ plants generally have higher *S*_C/O_ and lower *k* ^c^_cat_ then those from C_4_ plants.

Plants, algae, and many photosynthetic bacteria possess Form I Rubisco, which is made up of eight large subunits and eight small subunits (Tabita, Hanson, Satagopan, Witte, & Kreel, 2008). The crystal structures of several Form I Rubisco enzymes are known (Andersson & Backlund, 2008). The L_8_S_8_ holoenzyme has four L_2_ dimers, each with the two large subunits arranged head to tail and two active sites located at the interface between the two large subunits. Two groups of four small subunits interact with the four L_2_ dimers at the two ends, presumably to stabilize the four L_2_ dimers. Form I Rubisco can be further divided into green‐type (IA and IB) and red‐type Rubisco (IC and ID). The chloroplasts of plants and green algae contain Form IB Rubisco, which is assembled with the large subunits encoded by the *rbcL* gene of the plastome and the small subunits produced from a family of nuclear *RbcS* genes and transported into the chloroplast stroma. In contrast, Form ID Rubisco is found in the chloroplasts of red lineage and its subunits are encoded by genes in a single operon in chloroplast genomes (Tabita et al., 2008). Form ID Rubisco enzymes are typically associated with unusually large variation in their kinetic properties as demonstrated in a recent study of the Rubisco from diatoms (Young et al., 2016). Rubisco from a red alga *Griffithsia monilis* is especially of great biotechnological interest because it was reported to have a remarkably high *S*_C/O_ value of ~167 compared to a typical *S*_C/O_ value of ~ 92 in C_3_ crops, while its *k* ^c^_cat_ is similar to the average *k* ^c^_cat_ from C_3_ crops (Whitney, Baldet, Hudson, & Andrews, 2001; Zhu, Portis, & Long, 2004).

Despite significant technical advances achieved in recent decades, expressing a foreign Rubisco in plants remains challenging and is usually performed in tobacco, where chloroplast transformants can be readily generated (Sharwood, 2017; Whitney et al., 2011). Particularly, the folding of the Rubisco large subunit and the assembly of the L_8_S_8_ holoenzyme require multiple steps and a large number of specific protein chaperones (Bracher, Whitney, Hartl, & Hayer‐Hartl, 2017). As a result, previous attempts in Rubisco engineering were mostly limited to replacing the large subunit in tobacco with those from closely related plant species such as tomato and sunflower or simpler prokaryotic Rubisco from *Rhodospirillum rubrum* and *Methanococcoides burtonii* (Kanevski, Maliga, Rhoades, & Gutteridge, 1999; Whitney & Andrews, 2001; Wilson, Alonso, & Whitney, 2016; Zhang et al., 2011). In a previous attempt, the Rubisco subunits from a red alga *Galdieria sulphuraria* and a diatom *Phaeodactylum tricornutum* were unable to assemble into functional complexes in tobacco chloroplasts, likely because of their incompatibility with the folding and assembly factors of tobacco (Whitney et al., 2001). Recent identification and characterization of several Rubisco assembly factors such as BSD2, RBCX, RAF1, and RAF2 have greatly advanced our knowledge in Rubisco biogenesis at the molecular level and already improved the recombinant assembly of the Arabidopsis large subunit and the tobacco small subunit (Brutnell, Sawers, Mant, & Langdale, 1999; Feiz et al., 2012, 2014; Hauser et al., 2015; Liu et al., 2010; Saschenbrecker et al., 2007; Whitney, Birch, Kelso, Beck, & Kapralov, 2015). Recently, co‐expression of these assembly factors with chloroplast‐specific chaperonins led to the successful assembly of functional Arabidopsis and tobacco Rubisco enzymes in *E. coli* (Aigner et al., 2017). However, the biogenesis requirements of Rubisco from red algae are still not well understood.

We previously replaced the tobacco *rbcL* gene with genes encoding the large and small subunits from a cyanobacterium *Synechococcus elongatus* PCC7942, which were able to assemble functional L_8_S_8_ complex and support the phototrophic growth of the tobacco transformants (Lin, Occhialini, Andralojc, Parry, & Hanson, 2014; Occhialini, Lin, Andralojc, Hanson, & Parry, 2016). Those findings indicated that the folding and assembly requirements of cyanobacterial Rubisco are not as strict as the Rubisco from higher plants and can be readily met in tobacco. An extra C‐terminal extension on the small subunit of the red‐type Rubisco from *Rhodobacter sphaeroides* has recently been observed to mimic the role of assembly factors, RBCX and RAF1, which were absent in organisms with red‐type Rubisco, suggesting that red‐type Rubisco may not require specific assembly factors (Joshi, Mueller‐Cajar, Tsai, Hartl, & Hayer‐Hartl, 2015). Therefore, we decided to reassess the expression of red‐type Rubisco in higher plants. Our current work differs from the previous attempt to express the red‐type Rubisco in tobacco in two critical aspects: (i) We expressed the Rubisco subunits from the red alga *Griffithsia monilis*, whose Rubisco has a *k* ^c^_cat_ reported to be over twofold higher than that from *Galdieria sulphuraria,* and (ii) we replaced the tobacco *rbcL* gene with synthesized genes encoding the large and small subunits of *Griffithsia monilis*, whereas the subunits from *Galdieria sulphuraria* were previously expressed along with the native Rubisco subunits, which might have interfered with the assembly of the foreign Rubisco (Whitney et al., 2001). In addition, we incorporated the same arrangement of regulatory elements in intergenic regions that had previously been shown to be effective in expressing the cyanobacterial Rubisco subunits (Occhialini et al., 2016). Here, we report our latest attempt and relate our results with recent advances in Rubisco research.

## 2 METHODS

### 2.1 Construction of chloroplast transformation vectors

*Gm‐rbcL* and *Gm‐rbcS* genes encoding the Rubisco large and small subunits from *Griffithsia monilis* and *Gs‐cbbX* gene encoding the chloroplast Rubisco activase subunit from *Gracilaria salicornia* were optimized for codon usage in tobacco chloroplasts with OPTIMIZER (Puigbo, Guzman, Romeu, & Garcia‐Vallve, 2007) and synthesized by Bioneer Inc. (Alameda, CA). All oligonucleotides used in this work were ordered from Integrated DNA Technologies (Coralville, Iowa) and are listed in Table S1. Phusion High‐Fidelity DNA Polymerase from Thermo Fisher Scientific (Waltham, Massachusetts) was used for all DNA amplification steps. The FastDigest restriction enzymes and T4 DNA ligase from Thermo Fisher Scientific were used for all DNA cloning work. The sequencing of DNA constructs was performed at Biotechnology Resource Center at Cornell University.

*Gm‐rbcL* was amplified from the vector supplied by Bioneer Inc. with FL1‐GmrbcL5 and GmrbcLrev primers, which adds the 3′ end of the upstream chloroplast flanking region containing the native tobacco *rbcL* promoter at the 5′ end and a *Mau*BI restriction site at the 3′ end, respectively. For *Gm‐rbcL* ^*N*^, the FL1‐GmL2f primer was used instead of FL1‐GmrbcL5. The amplified *Gm‐rbcL* or *Gm‐rbcL* ^*N*^ gene was then joined with the upstream flanking region amplified from tobacco DNA extract with FL1f and FL1r primers using the overlap extension PCR procedure to generate the FL1‐Gm‐rbcL or FL1‐Gm‐rbcL^N^ DNA, which was then digested with *Cla*I and *Mau*BI and ligated into the similarly digested pCT‐rbcL vector (Lin et al., 2014). This replaced the cyanobacterial Rubisco large subunit gene in pCT‐rbcL vector with *Gm‐rbcL* or *Gm‐rbcL* ^*N*^ gene and generated the pCT‐Gm‐rbcL and pCT‐Gm‐rbcL^N^ vectors, which were then used to generate GmL and GmL^*N*^ tobacco chloroplast transformants, respectively.

The *Gm‐rbcS* and *Gs‐cbbX* gene modules were then added to the *Mau*BI site of the pCT‐Gm‐rbcL and pCT‐Gm‐rbcL^N^ vectors with a terminator, IEE, and ribosome binding site at their 5′ ends as previously described (Occhialini et al., 2016). Briefly, the gene modules were produced with the overlap extension PCR procedure using the primers listed in Table S1. The terminators At‐TrbcL and At‐TatpE were amplified from Arabidopsis DNA extract. Different combinations of four gene modules At‐TrbcL‐IE2‐SD‐Gm‐rbcS, At‐TatpE‐IEE‐SD‐Gm‐rbcS, At‐TrbcL‐IE2‐SD‐Gs‐cbbX, and At‐TatpE‐IEE‐SD‐Gs‐cbbX were added to the pCT‐Gm‐rbcL and pCT‐Gm‐rbcL^N^ vectors, and the resulting vectors were used to generate GmLS, GmLSX1, GmLSX2, and GmL^*N*^SX tobacco chloroplast transformants.

### 2.2 Generation of tobacco chloroplast transformants

The transformation of the tobacco chloroplast genome was performed as in our previous studies (Occhialini et al., 2016). Briefly, two‐week‐old tobacco (Nicotiana tabacum cv. Samsun) seedlings on MS agar medium were bombarded with 0.5 mg of 0.6 μm gold nanoparticles from Bio‐Rad Laboratories, Inc. (Hercules, CA) coated with 2 μg of each transformation vector in a Biolistic PDS‐1000/He Particle Delivery System from Bio‐Rad Laboratories, Inc. Eight to ten bombardments were performed for each construct. The seedlings were cut into pieces of about 25 mm^2^ and placed on RMOP agar medium with 500 mg/L spectinomycin for selection. The shoots arising after 4–6 weeks were then cut into pieces of about 5 mm^2^ and placed on the same RMOP agar medium with spectinomycin for another round of regeneration. The DNA samples were then extracted from the new shoots and checked for homoplasmy with a DIG‐labeled DNA probe on a DNA blot as described previously (Lin et al., 2014). The transformants that were homoplasmic were transferred to MS agar medium for rooting.

### 2.3 RNA blot analyses of the transplastomic tobacco plants

The DIG‐labeled RNA probe for each transgene (*Gm‐rbcL*,* Gm‐rbcS,* and *Gs‐cbbX*) was generated from DNA templates with T7 promoters and MEGAshortscript kit (Ambion, Foster City, CA) using the procedure described previously (Occhialini et al., 2016). RNA was extracted from leaf tissues (~ 2‐5 cm^2^) with PureLink^®^ RNA Mini Kit (Life Technologies) according to the manufacturer's protocol, and RNA concentrations were estimated with the Qubit^®^ RNA BR Assay Kit. Denaturing RNA gels with 2% formaldehyde were run with 0.2 μg of each RNA sample, transferred to a supercharged Nylon membrane in 20 x SSC buffer, hybridized with each DIG‐labeled RNA probe at 68°C for 12–16 hr, and detected with alkaline phosphatase‐conjugated anti‐Digoxigenin and CDP‐star chemiluminescent substrate from Roche Life Science (Pleasanton, California) as described previously (Occhialini et al., 2016).

### 2.4 Blue‐native PAGE, SDS‐PAGE, and immunoblots

Approximately 2‐5 cm^2^ of leaf tissues were ground in ice‐cold 150 μl of 100 mM Bicine pH8.0 with 10 mM MgCl_2_, 1 mM EDTA, 50 mM 2‐mercaptoethanol, 20 mM DTT, 20 mM NaHCO_3_, 2 mM Na_2_HPO_4_, and cOmplete Protease Inhibitor Cocktail Mini Tablet from Roche Life Science with a 2 ml Wheaton tissue homogenizer. The insoluble parts were separated by centrifugation at 16,000 × *g* for 5 min at 4°C and resuspended in 40 μl of 50 mM Tris‐HCl, 1 mM EDTA, 100 mM NaCl, 8 M urea pH 8.0 buffer. The protein concentrations were estimated by Bradford assay. For blue‐native PAGE, approximately 12 μg of total soluble proteins from each transformant was diluted to 50 mM Bis‐Tris, 50 mM NaCl, 0.001% Ponceau S, and 10% glycerol at pH 7.2 and loaded into a 3%–12% polyacrylamide gradient gel from Invitrogen (Carlsbad, CA) along with Novex NativeMark™ protein standard. The anode buffer was 50 mM bis‐Tris and 50 mM Tricine pH 6.8, and the cathode buffer contained additional 0.002% Coomassie Brilliant Blue G 250. The gels were run at 150 V for 30 min and then at 250 V for 1.5 hr at 4°C. The gel was either stained with Coomassie R‐250 or transferred to a PVDF membrane in 25 mM Tris base, 190 mM glycine, and 20% methanol at 100 V for 1 hr at 4°C. The Rubisco large subunits were detected with a primary antibody that recognizes homologs from multiple species including plants and cyanobacteria. The antibody was provided by Professor Martin Parry at Lancaster University. For SDS‐PAGE, 8–45 μg of insoluble proteins from each sample was loaded to Any kD™ Mini‐PROTEAN® TGX™ gel from Bio‐Rad along with 0.1 μg of total proteins (both soluble and insoluble) from the wild‐type sample as a control. After transferred to a nitrocellulose membrane, the membrane was temporarily stained with 0.1% Ponceau S solution, and the large subunits were detected with the same primary antibody.

### 2.5 GenBank accession numbers

**Table:** 

GmL	MG561426
GmLS	MG561427
GmLSX1	MG561428
GmLSX2	MG561429
GmL^*N*^	MG561430
GmL^*N*^SX	MG561431

## 3 RESULTS

### 3.1 The tobacco *rbcL* gene was replaced with the Rubisco genes from *Griffithsia monilis*

**Figure 1:**
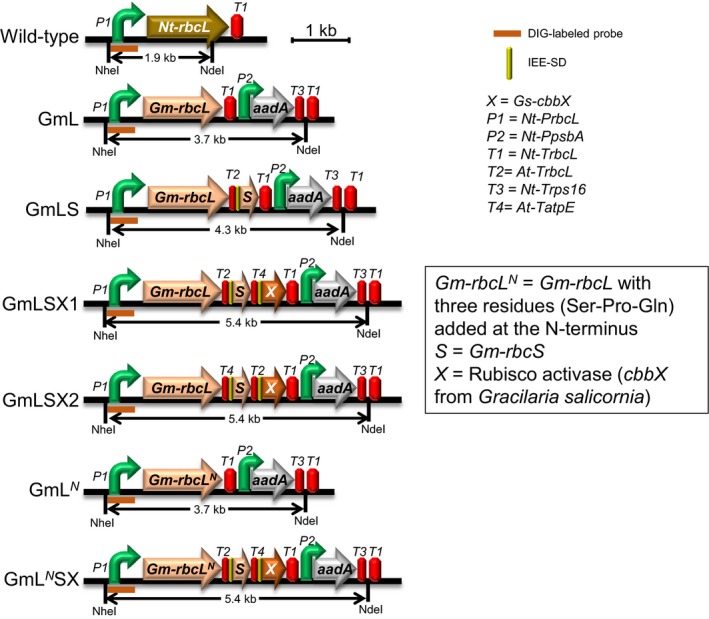
Comparison of the gene arrangements at the *rbcL* locus of different tobacco chloroplast transformants. The native *rbcL* gene was replaced in frame with *Gm‐rbcL* or *Gm‐rbc* *L*^*N*^ gene. The abbreviations of the regulatory elements and transgenes are defined on the right. The *Nhe*I and *Nde*I sites, the location of the DIG‐labeled DNA probe, and the size of the DNA fragment expected in DNA blot analysis are indicated in each construct

We replaced the *rbcL* gene from the tobacco chloroplast genome with different combinations of genes encoding Rubisco large and small subunits from *Griffithsia monilis* (GenBank accessions: ABU53651 and ABU53652) named *Gm‐rbcL* and *Gm‐rbcS*, respectively, and a Rubisco activase subunit (GenBank accession: YP_009019680) from a red alga *Gracilaria salicornia* named *Gs‐cbbX* using an approach described previously (Lin et al., 2014; Occhialini et al., 2016). Briefly, we synthesized the *Gm‐rbcL*,* Gm‐rbcS,* and *Gs‐cbbX* genes that are codon‐optimized for expression from tobacco chloroplast genome and replaced the entire coding region of the native *rbcL* gene with the *Gm‐rbcL* gene in frame. We also synthesized a modified *Gm‐rbcL* gene named *Gm‐rbcL* ^*N*^, which encodes three extra amino acid residues, Ser‐Pro‐Gln, at the N‐terminus that are highly conserved in the Rubisco large subunits of higher plants and known to be essential for the proper processing of their N‐termini (Figure S1) (Houtz, Magnani, Nayak, & Dirk, 2008). Figure 1 shows the gene arrangements in the *rbcL* locus of tobacco chloroplast genome in six different tobacco chloroplast transformants compared to that in the wild‐type tobacco chloroplast genome. In GmLS, GmLSX1, GmLSX2, and GmL^*N*^SX lines, a terminator, an intercistronic expression element (IEE), and a ribosome binding site or a Shine–Dalgarno sequence (SD) were inserted into the intergenic regions. We previously used the same arrangement of regulatory elements in intergenic regions to successfully express Rubisco genes from cyanobacterium *Synechococcus elongatus* PCC7942 from the tobacco chloroplast genome (Lin et al., 2014; Occhialini et al., 2016). The transgenes are followed by an additional operon containing the promoter and 5′‐UTR sequences of tobacco chloroplast *psbA* gene and an *aadA* selectable marker gene that encodes a streptomycin and spectinomycin resistance protein.

**Figure 2:**
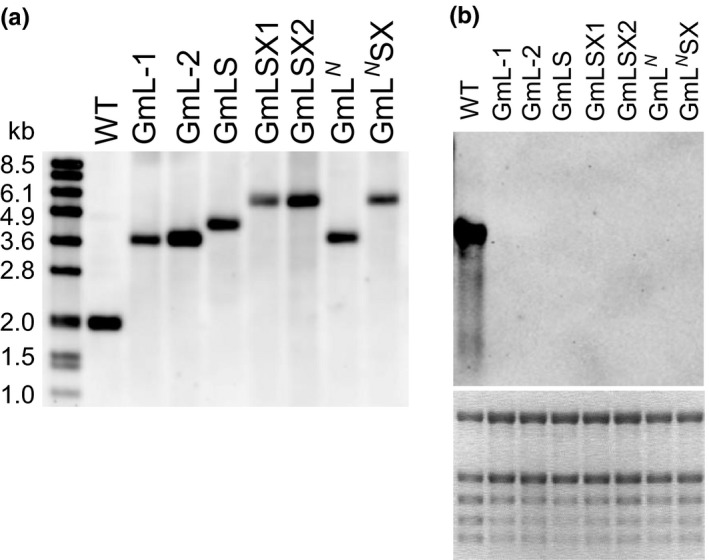
Confirmation of the complete deletion of the native *rbcL* gene in the tobacco chloroplast transformants with DNA and RNA blots. (a) DNA blot analysis of the *rbcL* locus using a DIG‐labeled probe shows that the transformants lack the DNA fragment at ~ 1.9 kb observed in the wild‐type (WT) sample and indicates that the *rbcL* locus is completely transformed in each transgenic plant. (b) RNA blot analysis using a DIG‐labeled RNA probe to detect the native *rbcL* transcript also confirms the absence of the native *rbcL* gene in the transformants. The bottom panel shows the ethidium bromide staining of the RNA gel to verify that comparable amounts of RNA were loaded for each sample

We identified the chloroplast transformants that are homoplasmic based on restriction fragment length polymorphism. After the DNA samples digested with *Nde*I and *Nhe*I restriction enzymes were separated on an agarose gel and blotted to a Nylon membrane, a DNA probe to detect the *rbcL* promoter region was used to determine the approximate lengths of the target DNA fragments. Figure 2a shows that each transformant has the fragment consistent with the expected length according to the transgenes introduced into the *rbcL* locus, and the fragment corresponding to that in the wild‐type sample is no longer present in the transformants, indicating that the *rbcL* locus is homoplasmic in these transformants. The complete deletion of the native *rbcL* gene in the transformants was also confirmed by the absence of its transcript on an RNA blot (Figure 2b). Although we initially obtained multiple independent homoplasmic lines from each transformation construct, many of them did not survive for prolonged period even after they were transplanted in fresh MS agar growth medium. Here, we present the results from two independent surviving lines of GmL transformants and one line from each of the other five transformants.

### 3.2 The *Griffithsia monilis* Rubisco genes produced transcripts in tobacco chloroplasts

**Figure 3:**
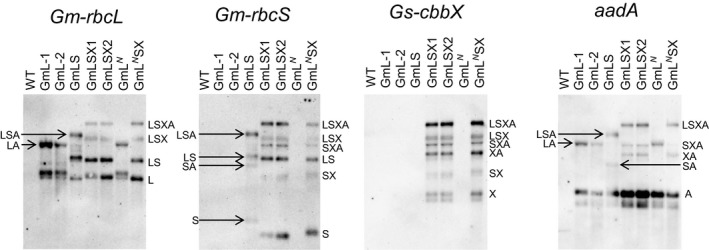
Expression of the transgenes in the tobacco chloroplast transformants analyzed by RNA blots. The four transgenes detected with the RNA probes are indicated on the top of each blot. The transcripts were assigned based on their sizes, and their names represent the order of the transgenes present in the transcripts with one‐letter abbreviations (L = *Gm‐rbcL*, S = *Gm‐rbcS*, X = *Gs‐cbbX* and A = *aadA*). Slight variations in sizes among similar transcripts (e.g., *Gm‐rbcS*) were due to their different terminators

We analyzed the transcript profiles in the transformants with RNA probes, each complementary to the transcripts arising from each of the *Gm‐rbcL*,* Gm‐rbcS*,* Gs‐cbbX*, and *aadA* transgenes. RNA blots show transcripts of multiple lengths were detected by each RNA probe (Figure 3). Each transformant produced transcripts in accordance with its transgenes. In addition to monocistronic transcripts, we are able to assign bands with higher molecular weights to polycistronic transcripts arising from either incomplete RNA processing at the IEE sites or read‐through transcription of the genes located downstream including *aadA*. These complex transcript profiles are similar to those in our previous study involving the same regulatory elements in intergenic regions (Occhialini et al., 2016).

### 3.3 The *Griffithsia monilis* Rubisco subunits failed to assemble into functional Rubisco in tobacco chloroplasts

**Figure 4:**
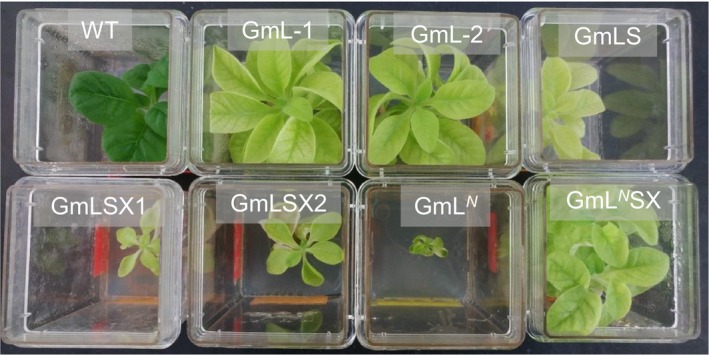
Phenotype of the tobacco transformants containing the *Griffithsia monilis* Rubisco genes grown in rooting medium containing sucrose

We transferred the homoplasmic shoots of each transformant to the rooting medium with sucrose and spectinomycin. Several of them grew poorly even in the growth medium containing sucrose, and they are very pale‐green compared to the wild‐type shoot (Figure 4). Previous studies of tobacco mutants lacking Rubisco also showed pale‐green leaves on sucrose‐containing medium and were unable to grow autotrophically (Kanevski et al., 1999; Whitney et al., 2001). The pale‐green phenotype of the transformants with the Rubisco genes from *Griffithsia monilis* suggested that they were also deficient of the Rubisco enzyme.

**Figure 5:**
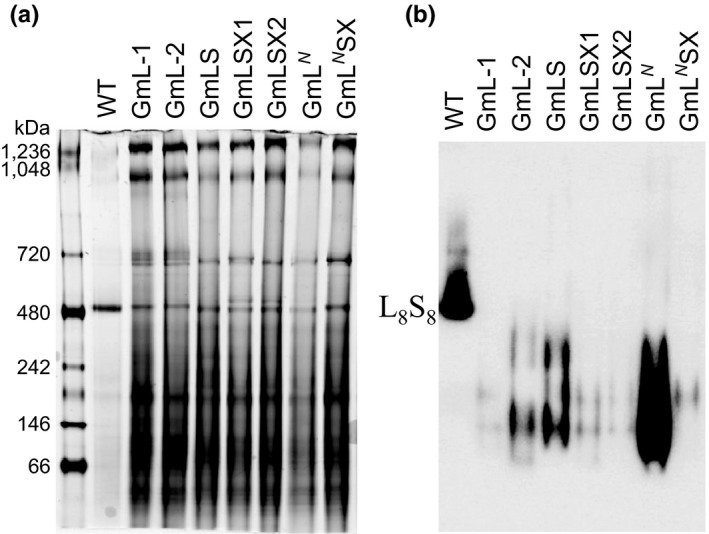
Native PAGE (polyacrylamide gel electrophoresis) analysis of the total soluble proteins (TSP) in the tobacco transformants. (a) Coomassie blue staining of Native PAGE with 2 μg of TSP from the wild‐type plant and 12 μg of TSP from each transformant. (b) The immunoblot of the Native PAGE with 0.1 μg of TSP from the wild‐type plant and 12 μg of TSP from each transformant. The Rubisco is detected by an antibody against the large subunit. The band corresponding to the L_8_S_8_ complex observed in the wild‐type sample is labeled

We extracted total soluble proteins from leaf tissues of the transformants and analyzed them on Native‐PAGE in order to determine whether the Rubisco subunits from *Griffithsia monilis* are able to assemble L_8_S_8_ complex in tobacco chloroplasts. Although a band close to the size of L_8_S_8_ was observed in the sample from each transformant when the gel was stained with Coomassie blue, an antibody for the large subunit was unable to detect any L_8_S_8_ complex in the transformants (Figure 5). Instead, smears at lower molecular weights were detected on the immunoblot of the Native PAGE. The signal from the GmL^*N*^ sample, which has the red algal large subunit with a modified N‐terminus, is much stronger, possibly due to improved translation efficiency or stability conferred by the modified N‐terminus.

**Figure 6:**
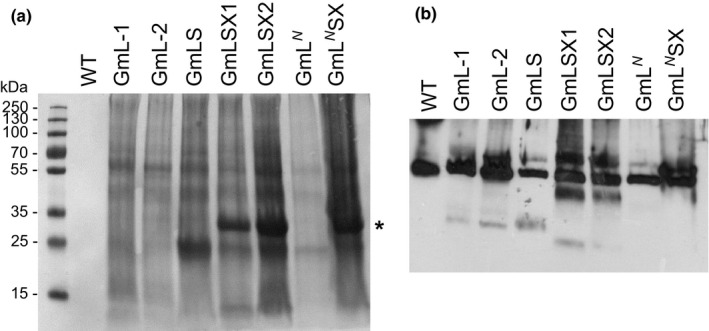
SDS‐PAGE analysis of the insoluble proteins in the tobacco transformants. For wild‐type, 0.1 μg of total proteins (both soluble and insoluble) was loaded, whereas for the transgenic lines, 8‐45 μg of insoluble proteins was loaded. (a) The proteins were transferred from PAGE to a nitrocellulose membrane and detected with Ponceau S staining. The 33 kDa band (marked with *) observed only in GmLSX1, GmLSX2, and GmL^*N*^SX is likely from the *Gracilaria salicornia* CbbX protein. (b) The blot was then used to detect the Rubisco large subunits with the same antibody used in Figure 5b

When the Rubisco subunits from another red algal species, *Galdieria sulphuraria*, were expressed in tobacco chloroplasts in a previous study, they were found to accumulate only in insoluble protein fractions (Whitney et al., 2001). SDS‐PAGE analysis of insoluble proteins from our transgenic lines indicates a faint band near 55 kDa, where the large subunits would be expected (Figure 6a). Subsequent immunoblotting confirms that the Rubisco large subunits were indeed present in the insoluble protein fractions (Figure 6b). We also observed a strong band near 33 kDa only in tobacco lines expressing plastid‐encoded CbbX from *Gracilaria salicorniai* (Figure 6a). This is consistent with a recent study, which found that the plastid‐encoded CbbX was prone to aggregation in the absence of its nuclear‐encoded partner (Loganathan, Tsai, & Mueller‐Cajar, 2016).

## 4 DISCUSSION

The Rubisco enzymes from red algal species are known to have the best CO_2_/O_2_ specificity factors while maintaining the catalytic rates high enough to potentially improve carbon fixation if they can be engineered into higher plants (Kanevski et al., 1999; Whitney et al., 2001; Zhu et al., 2004). Among them, the Rubisco from a red alga *Griffithsia monilis* was reported to have the best catalytic properties. In a previous attempt, the Rubisco subunits from another red alga *Galdieria sulphuraria* were produced in tobacco chloroplasts but failed to assemble into a functional L_8_S_8_ complex. Those transgenic plants still had the native Rubisco enzyme when the Rubisco genes from *Galdieria sulphuraria* were expressed (Whitney et al., 2001). As it is preferable to remove the native Rubisco, we replaced the native *rbcL* gene in the tobacco plastome with genes encoding the Rubisco subunits from *Griffithsia monilis* in our current work.

In addition to optimizing the codon usage of these genes, we also used the regulatory elements in the intergenic regions that had been previously shown to successfully express Rubisco subunits from a cyanobacterium *Synechococcus elongatus* PCC7942 in tobacco chloroplasts (Lin et al., 2014; Occhialini et al., 2016). However, none of the six combinations that we attempted produced functional *Griffithsia monilis* L_8_S_8_ complexes in tobacco chloroplasts. The transcript profiles from our current transgenic plants are complex due to incomplete processing at the IEE sites and inefficient transcription termination, but they are consistent with what we would expect from these regulatory elements. Presumably, the *Griffithsia monilis* Rubisco genes would be translated from these transcripts in a similar manner. In our opinion, the incompatibility of the Rubisco folding and assembly factors in tobacco with the *Griffithsia monilis* Rubisco subunits most likely caused the failure to assemble the *Griffithsia monilis* Rubisco enzyme.

Past and recent studies have made significant progress in unraveling the biogenesis for From I Rubisco (Bracher et al., 2017). Multiple proteins are known to be essential to overcome the high tendency of the large subunits to aggregate. These include BSD2 (Bundle Sheath Defective 2), which binds *rbcL* transcripts and possibly prevents the nascent large subunits from aggregation (Brutnell et al., 1999; Doron, Segal, Gibori, & Shapira, 2014) and chaperonin complexes made up of multiple Cpn60 homologs to assist the proper folding of the large subunits (Bai et al., 2015; Zhang et al., 2016) with the coordination of several co‐chaperonins such as Cpn10 and Cpn20 (Guo et al., 2015; Tsai, Mueller‐Cajar, Saschenbrecker, Hartl, & Hayer‐Hartl, 2012). However, the ability of these chaperonins to fold heterologous Rubisco large subunits is not well characterized.

Once the large subunit is properly folded, assembly factors such as RBCX, RAF1, and BSD2 help the transition from L_2_ dimers and L_8_ core to functional L_8_S_8_ complexes (Aigner et al., 2017; Feiz et al., 2012; Hauser et al., 2015; Kolesinski, Belusiak, Czarnocki‐Cieciura, & Szczepaniak, 2014; Kolesinski et al., 2013; Liu et al., 2010; Saschenbrecker et al., 2007). In a recent study, co‐expression of the large subunit and RAF1 from Arabidopsis in tobacco chloroplasts has shown that Rubisco from higher plants prefers native RAF1 for efficient assembly (Whitney et al., 2015). On the other hand, the red‐type Rubisco from *Rhodobacter sphaeroides* was able to assemble in *E. coli* in the absence of extra assembly factors, and evidence indicated that a C‐terminal extension in its small subunit plays a similar role as RBCX to achieve the efficient assembly of the L_8_S_8_ complex (Joshi et al., 2015). As a similar C‐terminal extension is also present in the small subunit of *Griffithsia monilis* (Figure S2), its Rubisco might also be able to assemble in a heterologous environment in a similar manner. If this is the case, the absence of *Griffithsia monilis* Rubisco in our transgenic plants is more likely due to the incompatibility between the large subunit from *Griffithsia monilis* and the tobacco Cpn60 chaperonins or other assembly factors such as BSD2 and RAF2. Recent success in heterologous assembly of plant Rubisco in *E. coli* highlights the multitude of folding and accessory proteins required in Rubisco biogenesis (Aigner et al., 2017). Clearly, further studies of chaperonins and assembly factors present in red algae are required to improve the heterologous expression of their Rubisco enzymes.

We have not modified the small subunit genes in our tobacco lines. Rubisco small subunits are known to form hybrid complexes with large subunits from other species (Kanevski et al., 1999; Zhang et al., 2011) and therefore could possibly interfere with proper assembly of red algal Rubisco. As it is likely that red algal large subunits failed to form a stable L_8_ core in our transgenic plants, they are not expected to interact with the tobacco small units. However, in the future, it would be preferable to engineer red algal Rubisco in plants lacking both large and small subunits to prevent their potential interference.

In our current work, we also expressed a slightly modified large subunit (*Gm‐rbcL* ^*N*^) from *Griffithsia monilis* by adding three residues to its N‐terminus (Ser‐Pro‐Gln) in order to match the N‐termini from the large subunits of higher plants, which are known to undergo highly conserved co‐ and post‐translational processing steps: the removal of the first two residues (Met‐Ser) and the acetylation of the third residue (Pro) (Houtz, Poneleit, Jones, Royer, & Stults, 1992). Previous work to replace the tobacco *rbcL* gene with foreign Rubisco genes often fused the first 11‐14 codons of the native *rbcL* gene to the foreign counterparts in order to preserve the high translation efficiency as well as the N‐terminus of the large subunit (Kanevski et al., 1999; Whitney & Andrews, 2001). Although the *Gm‐rbcL* ^*N*^ gene used in our study included the conserved proline residue at the N‐terminus for acetylation, we could not determine its acetylation status. Further studies in the general motif at the N‐terminus and its processing enzymes are needed to determine the factors necessary for proper N‐terminal processing of foreign Rubisco large subunits.

The active site of Rubisco is prone to inhibition by a number of sugar phosphates including its natural substrate RuBP, misfired products such as Xylulose‐1,5‐bisphosphate, and natural inhibitors such as 2‐carboxy‐d‐arabinitol 1‐phosphate. The inhibited Rubisco must be activated by another chaperone known as Rubisco activase (Mueller‐Cajar, Stotz, & Bracher, 2014). Although both the green‐type and red‐type Rubisco activases belong to the AAA+ protein family, they have evolved independently from different gene families, and structural studies have revealed significant differences in their mechanisms to remove inhibitors from the Rubisco active sites (Mueller‐Cajar et al., 2011; Stotz et al., 2011). In this work, we co‐expressed a Rubisco activase gene, *cbbX*, from the chloroplast of a red alga *Gracilaria salicornia* with the Rubisco genes from *Griffithsia monilis* in tobacco chloroplasts. As the *cbbX* sequences from *Griffithsia monilis* are not available yet, we used a homolog from *Gracilaria salicornia* because of the high similarity of the Rubisco large subunits (95% identity) between the two species. Red algae possess two *cbbX* genes, one in the nuclear genomes and one in the plastid genomes. Recently, it was demonstrated that plastid‐encoded CbbX is prone to aggregation, and both gene products are required to form a hetero‐oligomeric complex to be functional (Loganathan et al., 2016). In our transgenic plants, we also observed the plastid‐encoded CbbX mostly in the insoluble protein fractions. Thus, future attempts to express Rubisco activase from red algae will certainly necessitate both nuclear and plastid *cbbX* genes.

In summary, our attempt to engineer the red‐type Rubisco from *Griffithsia monilis* in tobacco chloroplasts did not produce functional L_8_S_8_ Rubisco enzyme despite recent work suggesting that a bacterial red‐type Rubisco may not require extra assembly factors. Our hypothesis is that the folding chaperonins of tobacco chloroplasts are not compatible with the large subunit from *Griffithsia monilis* to produce properly folded L_2_ dimer intermediate form, and as a result, both the large and small subunits of *Griffithsia monilis* are degraded. Unlike eukaryotic Rubisco, the Rubisco from prokaryotes such as *Rhodospirillum rubrum*,* Methanococcoides burtonii*, and *Synechococcus elongatus* PCC7942 can readily be expressed in tobacco chloroplasts as well as in *E. coli* (Lin et al., 2014; Whitney & Andrews, 2001; Wilson et al., 2016). The specific folding and assembly requirements of eukaryotic L_8_S_8_ Rubisco enzymes still present a major challenge in improving the activity of Rubisco in higher plants.

## AUTHOR CONTRIBUTIONS

M.T.L. performed the experiments. M.R.H. and M.T.L. designed the experiments, analyzed the data, and wrote the manuscript.

## References

[pld345-bib-0001] Aigner, H. , Wilson, R. H. , Bracher, A. , Calisse, L. , Bhat, J. Y. , Hartl, F. U. , & Hayer‐Hartl, M. (2017). Plant RuBisCo assembly in *E. coli* with five chloroplast chaperones including BSD2. Science, 358, 1272–1278. 10.1126/science.aap9221 29217567

[pld345-bib-0002] Andersson, I. , & Backlund, A. (2008). Structure and function of Rubisco. Plant Physiology and Biochemistry, 46, 275–291. 10.1016/j.plaphy.2008.01.001 18294858

[pld345-bib-0003] Bai, C. C. , Guo, P. , Zhao, Q. , Lv, Z. Y. , Zhang, S. J. , Gao, F. , … Liu, C. (2015). Protomer roles in chloroplast chaperonin assembly and function. Molecular Plant, 8, 1478–1492. 10.1016/j.molp.2015.06.002 26057234

[pld345-bib-0004] Bracher, A. , Whitney, S. M. , Hartl, F. U. , & Hayer‐Hartl, M. (2017). Biogenesis and metabolic maintenance of Rubisco. Annual Review of Plant Biology, 68, 29–60. 10.1146/annurev-arplant-043015-111633 28125284

[pld345-bib-0005] Brutnell, T. P. , Sawers, R. J. , Mant, A. , & Langdale, J. A. (1999). BUNDLE SHEATH DEFECTIVE2, a novel protein required for post‐translational regulation of the *rbcL* gene of maize. The Plant Cell, 11, 849–864. 10.1105/tpc.11.5.849 10330470 PMC144220

[pld345-bib-0006] Doron, L. , Segal, N. , Gibori, H. , & Shapira, M. (2014). The BSD2 ortholog in *Chlamydomonas reinhardtii* is a polysome‐associated chaperone that co‐migrates on sucrose gradients with the *rbcL* transcript encoding the Rubisco large subunit. The Plant Journal, 80, 345–355. 10.1111/tpj.12638 25124725

[pld345-bib-0007] Feiz, L. , Williams‐Carrier, R. , Belcher, S. , Montano, M. , Barkan, A. , & Stern, D. B. (2014). A protein with an inactive pterin‐4a‐carbinolamine dehydratase domain is required for Rubisco biogenesis in plants. The Plant Journal, 80, 862–869. 10.1111/tpj.12686 25279696

[pld345-bib-0008] Feiz, L. , Williams‐Carrier, R. , Wostrikoff, K. , Belcher, S. , Barkan, A. , & Stern, D. B. (2012). Ribulose‐1,5‐bis‐phosphate carboxylase/oxygenase accumulation factor1 is required for holoenzyme assembly in maize. The Plant Cell, 24, 3435–3446. 10.1105/tpc.112.102012 22942379 PMC3462642

[pld345-bib-0009] Guo, P. , Jiang, S. , Bai, C. C. , Zhang, W. J. , Zhao, Q. , & Liu, C. M. (2015). Asymmetric functional interaction between chaperonin and its plastidic cofactors. FEBS Journal, 282, 3959–3970. 10.1111/febs.13390 26237751

[pld345-bib-0010] Hauser, T. , Bhat, J. Y. , Milicic, G. , Wendler, P. , Hartl, F. U. , Bracher, A. , & Hayer‐Hartl, M. (2015). Structure and mechanism of the Rubisco‐assembly chaperone Raf1. Nature Structural & Molecular Biology, 22, 720–728. 10.1038/nsmb.3062 26237510

[pld345-bib-0011] Houtz, R. L. , Magnani, R. , Nayak, N. R. , & Dirk, L. M. (2008). Co‐ and post‐translational modifications in Rubisco: Unanswered questions. Journal of Experimental Botany, 59, 1635–1645. 18353761 10.1093/jxb/erm360

[pld345-bib-0012] Houtz, R. L. , Poneleit, L. , Jones, S. B. , Royer, M. , & Stults, J. T. (1992). Posttranslational modifications in the amino‐terminal region of the large subunit of ribulose‐1,5‐bisphosphate carboxylase/oxygenase from several plant species. Plant Physiology, 98, 1170–1174. 10.1104/pp.98.3.1170 16668742 PMC1080323

[pld345-bib-0013] Joshi, J. , Mueller‐Cajar, O. , Tsai, Y. C. , Hartl, F. U. , & Hayer‐Hartl, M. (2015). Role of small subunit in mediating assembly of red‐type form I Rubisco. Journal of Biological Chemistry, 290, 1066–1074. 10.1074/jbc.M114.613091 25371207 PMC4294474

[pld345-bib-0014] Kanevski, I. , Maliga, P. , Rhoades, D. F. , & Gutteridge, S. (1999). Plastome engineering of ribulose‐1,5‐bisphosphate carboxylase/oxygenase in tobacco to form a sunflower large subunit and tobacco small subunit hybrid. Plant Physiology, 119, 133–142. 10.1104/pp.119.1.133 9880354 PMC32212

[pld345-bib-0015] Kolesinski, P. , Belusiak, I. , Czarnocki‐Cieciura, M. , & Szczepaniak, A. (2014). Rubisco Accumulation Factor 1 from *Thermosynechococcus elongatus* participates in the final stages of ribulose‐1,5‐bisphosphate carboxylase/oxygenase assembly in *Escherichia coli* cells and in vitro. FEBS Journal, 281, 3920–3932. 10.1111/febs.12928 25041569

[pld345-bib-0016] Kolesinski, P. , Golik, P. , Grudnik, P. , Piechota, J. , Markiewicz, M. , Tarnawski, M. , … Szczepaniak, A. (2013). Insights into eukaryotic Rubisco assembly ‐ Crystal structures of RbcX chaperones from *Arabidopsis thaliana* . Biochimica et Biophysica Acta, 1830, 2899–2906. 10.1016/j.bbagen.2012.12.025 23295968

[pld345-bib-0017] Kromdijk, J. , Glowacka, K. , Leonelli, L. , Gabilly, S. T. , Iwai, M. , Niyogi, K. K. , & Long, S. P. (2016). Improving photosynthesis and crop productivity by accelerating recovery from photoprotection. Science, 354, 857–861. 10.1126/science.aai8878 27856901

[pld345-bib-0018] Lin, M. T. , Occhialini, A. , Andralojc, P. J. , Parry, M. A. , & Hanson, M. R. (2014). A faster Rubisco with potential to increase photosynthesis in crops. Nature, 513, 547–550. 10.1038/nature13776 25231869 PMC4176977

[pld345-bib-0019] Liu, C. , Young, A. L. , Starling‐Windhof, A. , Bracher, A. , Saschenbrecker, S. , Rao, B. V. , … Hayer‐Hartl, M. (2010). Coupled chaperone action in folding and assembly of hexadecameric Rubisco. Nature, 463, 197–202. 10.1038/nature08651 20075914

[pld345-bib-0020] Loganathan, N. , Tsai, Y. C. , & Mueller‐Cajar, O. (2016). Characterization of the heterooligomeric red‐type rubisco activase from red algae. Proceedings of the National Academy of Sciences of the United States of America, 113, 14019–14024. 10.1073/pnas.1610758113 27872295 PMC5150372

[pld345-bib-0021] Long, S. P. , Zhu, X. G. , Naidu, S. L. , & Ort, D. R. (2006). Can improvement in photosynthesis increase crop yields? Plant, Cell and Environment, 29, 315–330. 10.1111/j.1365-3040.2005.01493.x 17080588

[pld345-bib-0022] Mueller‐Cajar, O. , Stotz, M. , & Bracher, A. (2014). Maintaining photosynthetic CO_2_ fixation via protein remodelling: The Rubisco activases. Photosynthesis Research, 119, 191–201. 10.1007/s11120-013-9819-0 23543331

[pld345-bib-0023] Mueller‐Cajar, O. , Stotz, M. , Wendler, P. , Hartl, F. U. , Bracher, A. , & Hayer‐Hartl, M. (2011). Structure and function of the AAA+ protein CbbX, a red‐type Rubisco activase. Nature, 479, 194–199. 10.1038/nature10568 22048315

[pld345-bib-0024] Occhialini, A. , Lin, M. T. , Andralojc, P. J. , Hanson, M. R. , & Parry, M. A. J. (2016). Transgenic tobacco plants with improved cyanobacterial Rubisco expression but no extra assembly factors grow at near wild‐type rates if provided with elevated CO_2_ . The Plant Journal, 85, 148–160. 10.1111/tpj.13098 26662726 PMC4718753

[pld345-bib-0025] Ort, D. R. , Merchant, S. S. , Alric, J. , Barkan, A. , Blankenship, R. E. , Bock, R. , … Zhu, X. G. (2015). Redesigning photosynthesis to sustainably meet global food and bioenergy demand. Proceedings of the National Academy of Sciences of the United States of America, 112, 8529–8536. 10.1073/pnas.1424031112 26124102 PMC4507207

[pld345-bib-0026] Puigbo, P. , Guzman, E. , Romeu, A. , & Garcia‐Vallve, S. (2007). OPTIMIZER: A web server for optimizing the codon usage of DNA sequences. Nucleic Acids Research, 35, W126–131. 10.1093/nar/gkm219 17439967 PMC1933141

[pld345-bib-0027] Saschenbrecker, S. , Bracher, A. , Rao, K. V. , Rao, B. V. , Hartl, F. U. , & Hayer‐Hartl, M. (2007). Structure and function of RbcX, an assembly chaperone for hexadecameric Rubisco. Cell, 129, 1189–1200. 10.1016/j.cell.2007.04.025 17574029

[pld345-bib-0028] Savir, Y. , Noor, E. , Milo, R. , & Tlusty, T. (2010). Cross‐species analysis traces adaptation of Rubisco toward optimality in a low‐dimensional landscape. Proceedings of the National Academy of Sciences of the United States of America, 107, 3475–3480. 10.1073/pnas.0911663107 20142476 PMC2840432

[pld345-bib-0029] Sharwood, R. E. (2017). Engineering chloroplasts to improve Rubisco catalysis: Prospects for translating improvements into food and fiber crops. New Phytologist, 213, 494–510. 10.1111/nph.14351 27935049

[pld345-bib-0030] Simkin, A. J. , Lopez‐Calcagno, P. E. , Davey, P. A. , Headland, L. R. , Lawson, T. , Timm, S. , … Raines, C. A. (2017). Simultaneous stimulation of sedoheptulose 1,7‐bisphosphatase, fructose 1,6‐bisphophate aldolase and the photorespiratory glycine decarboxylase‐H protein increases CO_2_ assimilation, vegetative biomass and seed yield in Arabidopsis. Plant Biotechnology Journal, 15, 805–816. 10.1111/pbi.12676 27936496 PMC5466442

[pld345-bib-0031] Stotz, M. , Mueller‐Cajar, O. , Ciniawsky, S. , Wendler, P. , Hartl, F. U. , Bracher, A. , & Hayer‐Hartl, M. (2011). Structure of green‐type Rubisco activase from tobacco. Nature Structural & Molecular Biology, 18, 1366–1370. 10.1038/nsmb.2171 22056769

[pld345-bib-0032] Tabita, F. R. , Hanson, T. E. , Satagopan, S. , Witte, B. H. , & Kreel, N. E. (2008). Phylogenetic and evolutionary relationships of RubisCO and the RubisCO‐like proteins and the functional lessons provided by diverse molecular forms. Philosophical Transactions of the Royal Society of London. Series B, Biological sciences, 363, 2629–2640. 10.1098/rstb.2008.0023 18487131 PMC2606765

[pld345-bib-0033] Tcherkez, G. G. B. , Farquhar, G. D. , & Andrews, T. J. (2006). Despite slow catalysis and confused substrate specificity, all ribulose bisphosphate carboxylases may be nearly perfectly optimized. Proceedings of the National Academy of Sciences of the United States of America, 103, 7246–7251. 10.1073/pnas.0600605103 16641091 PMC1464328

[pld345-bib-0034] Tsai, Y. C. C. , Mueller‐Cajar, O. , Saschenbrecker, S. , Hartl, F. U. , & Hayer‐Hartl, M. (2012). Chaperonin cofactors, Cpn10 and Cpn20, of green algae and plants function as hetero‐oligomeric ring complexes. Journal of Biological Chemistry, 287, 20471–20481. 10.1074/jbc.M112.365411 22518837 PMC3370230

[pld345-bib-0035] Whitney, S. M. , & Andrews, T. J. (2001). Plastome‐encoded bacterial ribulose‐1,5‐bisphosphate carboxylase/oxygenase (RubisCO) supports photosynthesis and growth in tobacco. Proceedings of the National Academy of Sciences of the United States of America, 98, 14738–14743. 10.1073/pnas.261417298 11724961 PMC64751

[pld345-bib-0036] Whitney, S. M. , Baldet, P. , Hudson, G. S. , & Andrews, T. J. (2001). Form I Rubiscos from non‐green algae are expressed abundantly but not assembled in tobacco chloroplasts. The Plant Journal, 26, 535–547. 10.1046/j.1365-313x.2001.01056.x 11439139

[pld345-bib-0037] Whitney, S. M. , Birch, R. , Kelso, C. , Beck, J. L. , & Kapralov, M. V. (2015). Improving recombinant Rubisco biogenesis, plant photosynthesis and growth by coexpressing its ancillary RAF1 chaperone. Proceedings of the National Academy of Sciences of the United States of America, 112, 3564–3569. 10.1073/pnas.1420536112 25733857 PMC4371954

[pld345-bib-0038] Whitney, S. M. , Houtz, R. L. , & Alonso, H. (2011). Advancing our understanding and capacity to engineer nature's CO_2_‐sequestering enzyme, rubisco. Plant Physiology, 155, 27–35. 10.1104/pp.110.164814 20974895 PMC3075749

[pld345-bib-0039] Wilson, R. H. , Alonso, H. , & Whitney, S. M. (2016). Evolving *Methanococcoides burtonii* archaeal Rubisco for improved photosynthesis and plant growth. Scientific Reports, 6, 22284 10.1038/srep22284 26926260 PMC4772096

[pld345-bib-0040] Young, J. N. , Heureux, A. M. C. , Sharwood, R. E. , Rickaby, R. E. M. , Morel, F. M. M. , & Whitney, S. M. (2016). Large variation in the Rubisco kinetics of diatoms reveals diversity among their carbon‐concentrating mechanisms. Journal of Experimental Botany, 67, 3445–3456. 10.1093/jxb/erw163 27129950 PMC4892730

[pld345-bib-0041] Zhang, X. H. , Webb, J. , Huang, Y. H. , Lin, L. , Tang, R. S. , & Liu, A. (2011). Hybrid Rubisco of tomato large subunits and tobacco small subunits is functional in tobacco plants. Plant Science, 180, 480–488. 10.1016/j.plantsci.2010.11.001 21421395

[pld345-bib-0042] Zhang, S. J. , Zhou, H. , Yu, F. , Bai, C. C. , Zhao, Q. , He, J. H. , & Liu, C. M. (2016). Structural insight into the cooperation of chloroplast chaperonin subunits. BMC Biology, 14, 29 10.1186/s12915-016-0251-8 27072913 PMC4828840

[pld345-bib-0043] Zhu, X. G. , de Sturler, E. , & Long, S. P. (2007). Optimizing the distribution of resources between enzymes of carbon metabolism can dramatically increase photosynthetic rate: A numerical simulation using an evolutionary algorithm. Plant Physiology, 145, 513–526. 10.1104/pp.107.103713 17720759 PMC2048738

[pld345-bib-0044] Zhu, X. G. , Portis, A. R. , & Long, S. P. (2004). Would transformation of C_3_ crop plants with foreign Rubisco increase productivity? A computational analysis extrapolating from kinetic properties to canopy photosynthesis. Plant, Cell and Environment, 27, 155–165. 10.1046/j.1365-3040.2004.01142.x

