# Supplementary material for: Red algal Rubisco fails to accumulate in transplastomic tobacco expressing Griffithsia monilis RbcL and RbcS genes
Source: Plant Direct. 2018 Feb 28;2(2):e00045. doi: 10.1002/pld3.45 (PMC6508576; doi:10.1002/pld3.45)
Supplement: Supplementary file 1 [file PLD3-2-e00045-s001.pdf]

```
Tobacco_LSU      MSPQTETK---ASVGFKAGVK-EYKLTYYTPEYQTKDTDILAAFRVTPQPGVPPEEAGAA   56
Gm_LSU           MSNSVEERTRIKNERYESGVIPYAKMGYWDPNYAVKDTDILALFRVSPQPGVDPVEASAA   60
                 ** ..* :    .  :::**    *: *: *:* .******* ***:***** * **.**

Tobacco_LSU      VAAESSTGTWTTVWTDGLTSLDRYKGRCYRIERVVGEKDQYIAYVAYPLDLFEEGSVTNM   116
Gm_LSU           VAGESSTATWTVVWTDLLTACDLYRAKAYKVESVPNTSDQYFAYISYDIDLFEEGSIANL   120
                 **.****.***.**** **: * *:.:.*::* * . .***:**::* :*******::*:

Tobacco_LSU      FTSIVGNVFGFKALRALRLEDLRIPPAYVKTFQGPPHGIQVERDKLNKYGRPLLGCTIKP   176
Gm_LSU           TASIIGNVFGFKAVKALRLEDMRIPVAYLKTFQGPATGIVVERERMDKFGRPFLGATVKP   180
                  :**:********::******:*** **:******  ** ***::::*:***:**.*:**

Tobacco_LSU      KLGLSAKNYGRAVYECLRGGLDFTKDDENVNSQPFMRWRDRFLFCAEALYKAQAETGEIK   236
Gm_LSU           KLGLSGKNYGRVVYEGLRGGLDFLKDDENINSQPFMRWKERFLYSIEAVNRSIAATGEVK   240
                 *****.*****.*** ******* *****:********::***:. **: :: * ***:*

Tobacco_LSU      GHYLNATAGTCEEMIKRAVFARELGVPIVMHDYLTGGFTANTSLAHYCRDNGLLLHIHRA   296
Gm_LSU           GHYMNVTAATMEEMYERAEFAKQLGTVIIMIDLVI-GYTAIQTMGIWARKNDMILHLHRA   299
                 ***:*.**.* *** :** **::**. *:* * :  *:**  ::. :.*.*.::**:***

Tobacco_LSU      MHAVIDRQKNHGIHFRVLAKALRMSGGDHIHSGTVVGKLEGERDITLGFVDLLRDDFVEQ   356
Gm_LSU           GNSTYSRQKIHGMNFRVICKWMRMAGVDHIHAGTVVGKLEGDPLMIRGFYNTLLLPYLEV   359
                  ::. .*** **::***:.* :**:* ****:*********:  :  ** : *   ::*

Tobacco_LSU      DRSRGIYFTQDWVSLPGVLPVASGGIHVWHMPALTEIFGDDSVLQFGGGTLGHPWGNAPG   416
Gm_LSU           NLPQGIFFQQDWASLRKVTPVASGGIHCGQMHQLLDYLGNDVVLQFGGGTIGHPDGIQAG   419
                 :  :**:* ***.**  *  ********  :*  * : :*:* ********:*** *   *

Tobacco_LSU      AVANRVALEACVKARNEGRDLAQEGNEIIREACKWSPELAAACEVWKEIVFNFAAVDVLD   476
Gm_LSU           ATANRVALESMVIARNEGRDYVAEGPQILRDAAKTCGPLQTALDLWKDITFNYTSTDTAD   479
                 *.*******: * ******* . ** :*:*:*.* .  * :* ::**:*.**:::.*. *

Tobacco_LSU      K--------      477
Gm_LSU           FVETPTANV      488
```

Figure S1. Multiple sequence alignment of the Rubisco large subunits from tobacco and *Griffithsia monilis* using Clustal Omega version 1.2.4 (https://www.ebi.ac.uk/Tools/msa/clustalo/). Identical (*), conservative (:) and semi-conservative (.) residues are marked below the sequences.

```
Gm_SSU      -------MRLTQGTFSFLPDLTDEQIKKQVDYAISQNWAINIEYTEDP------------        41
Rs_SSU      -------MRITQGCFSFLPDLTDEQISAQVDYCLGRGWAVSLEHTDDP------------        41
Nt_SSU      MQVWPPINKKKYETLSYLPDLSQEQLLSEVEYLLKNGWVPCLEFETEHGFVYRENNKSPG        60
Se_SSU      MSMKTLPKERRFETFSYLPPLSDRQIAAQIEYMIEQGFHPLIEFNEHS------------        48
                    .     :*:** *::.*:  :::* : ..:   :*.  .

Gm_SSU      HPRNNFWELWGLPLFDINDAATVMYEIGSCRQQHSNVYIKVNAFDNTRGVESCVLSFLIN        101
Rs_SSU      HPRNTYWEMWGMPMFDLRDPKGVMIELDECRKAWPGRYIRINAFDSTRGFETVTMSFIVN        101
Nt_SSU      YYDGRYWTMWKLPMFGCTDATQVLAEVEEAKKAYPQAWIRIIGFDNVRQVQC--ISFIAY        118
Se_SSU      NPEEFYWTMWKLPLFDCKSPQQVLDEVRECRSEYGDCYIRVAGFDNIKQCQT--VSFIVH        106
                 :* :* :*:*.  .   *: *: ..:.     :*:: .**. :  :   :**:

Gm_SSU      RPSYEPGFRLVRSEDISRNQKYSFHSYATDKPEGSRY       138
Rs_SSU      RPEVEPSLRMERTEVDGRSIRYTHSIVR---------       129
Nt_SSU      KPEGY--------------------------------       123
Se_SSU      RPGRY--------------------------------       111
            :*
```

Figure S2. Multiple sequence alignment of the Rubisco small subunits from *Griffithsia monilis* (Gm_SSU), *Rhodobacter sphaeroides* (Rs_SSU), tobacco (Nt_SSU) and *Synechococcus elongatus* PCC6301 (Se_SSU) using Clustal Omega version 1.2.4 (https://www.ebi.ac.uk/Tools/msa/clustalo/). Identical (*), conservative (:) and semi-conservative (.) residues are marked below the sequences.
