# Supplementary material for: Red algal Rubisco fails to accumulate in transplastomic tobacco expressing Griffithsia monilis RbcL and RbcS genes
Source: Plant Direct. 2018 Feb 28;2(2):e00045. doi: 10.1002/pld3.45 (PMC6508576; doi:10.1002/pld3.45)
Supplement: Supplementary file 3 [file PLD3-2-e00045-s003.pdf]

Supplemental Data

Table S1. Oligonucleotides used in the construction and sequencing of the transformation vectors and generation of RNA probes. The MluI and MauBI restriction sites are underlined.

Name	Sequence
FL1f	CACTATCTCGACCTTGAACTAC
FL1r	AAATCCCTCCCTACAACTCATG
FL1-GmrbcL5	CATGAGTTGTAGGGAGGGATTTATGAGTAACTCTGTTGAGGAAAG
FL1-GmL2f	CATGAGTTGTAGGGAGGGATTTATGTCACCACAAAGTAACTCTGTTGAGGAAAGAAC
GmrbcLrev	AGATCGCGCGCGTTACACATTAGCTGTTGGTGTTTC
GmrbcLseq3	ACATTTCTTCCATTGTAGCTGCT
GmrbcLseq5	AGCTGTAAATAGATCTATTGCTGCA
TrbcLAtF	AGATGGGCCCACGCGTCGCGCGCGTGAATTAGATTTAGTAATTCACGTTTG
TrbcLAt-IE2R	CCATTCCGTTGTAAATAAATGATTTTAACAACAGATAACCTCGGCTCAATC
TatpE-At5	AGATGGGCCCACGCGTCGCGCGCGTTTTGTAACTAGCTGACGTGTAAAAAAAAAG
TatpE-At-IEE3	CCATTCCGTTGTAAATAAATGATCTTATTGGTTCAAATCCAATAGTAGGTATAAC
IE2-SD3	GTATATCTCCTTCTTGAGGTCTGTTGACTTTGTATACCATTCCGTTGTAAATAAATGATT
IEE-SD3	GTATATCTCCTTCTTGAGATCTGTTGACTTTGTATACCATTCCGTTGTAAATAAATGATC
IEESD-GmSF	GTCAACAGATCTCAAGAAGGAGATATACCCATGCGATTAACTCAAGGCACATTC
MluI-GmSR	AGATACGCGTTTAATATCTTGACCCTTCAGGCTTATC
IEESD-GsXF	GTCAACAGATCTCAAGAAGGAGATATACCCATGATTGACAATACTTTAGTTAACTTGC
MluI-GsXR	AGATACGCGTTTATTGAGTAAACAAGCGGCTTTTC
GsXseq3	CAACTAATAGTAAAGCGGCTATTTC
T7-GmLR	GAAATTAATACGACTCACTATAGGGTTACACATTAGCTGTTGGTGTTTC
T7-GmSR	GAAATTAATACGACTCACTATAGGGTTAATATCTTGACCCTTCAGGCTTATC
T7-GsXR	GAAATTAATACGACTCACTATAGGGTTATTGAGTAAACAAGCGGCTTTTC
