# Supplementary material for: Red algal Rubisco fails to accumulate in transplastomic tobacco expressing Griffithsia monilis RbcL and RbcS genes
Source: Plant Direct. 2018 Feb 28;2(2):e00045. doi: 10.1002/pld3.45 (PMC6508576; doi:10.1002/pld3.45)
Supplement: Supplementary file 4 [file PLD3-2-e00045-s004.pdf]

Reviewer #1

Comments for the authors to consider:

1. The recent breakthrough report on the expression of Arabidopsis thaliana RuBisCo in E. coli should be cited (Aigner H et al. (2017) Science 358: 1272-1278). The message of the paper is that co-expression of five chaperones was necessary to achieve assembly of the large and small subunits.

Authors' response: We added citations of (Aigner H et al. (2017) Science 358: 1272-1278) in the fourth paragraph of the "Introduction" section and the fourth paragraph of the "Discussion" section. Our manuscript was submitted before the Aigner et al paper appeared.

2. Inefficient transcription termination and incomplete mRNA processing could be pointed out as an explanation for complex mRNA profile.

Authors' response: In the second part of the "Results" section, we mentioned the cause of polycistronic transcripts as "incomplete RNA processing at the IEE sites or read-through transcription of the genes located downstream including *aadA*". We also added the following explanation in the second paragraph of the "Discussion" section: "The transcript profiles from our current transgenic plants are complex due to incomplete processing at the IEE sites and inefficient transcription termination …".

3. Interference with unassembled tobacco RuBisCo small subunit could also be pointed out as the potential problem with Griffithsia monilis RuBisCo assembly.

Authors' response: We added the following paragraph to Discussion section: "We have not modified the small subunit genes in our tobacco lines. Rubisco small subunits are known to form hybrid complexes with large subunits from other species (Kanevski et al., 1999; Zhang et al., 2011), and therefore could possibly interfere with proper assembly of red algal Rubisco. Since it is likely that red algal large subunits failed to form stable L_8 core in our transgenic plants, they are not expected to interact with the tobacco small units. However, it would be preferable to engineer red algal Rubisco in the future in plants lacking both large and small subunits to prevent their potential interference."

Reviewer #2

The manuscript deals in functional expression of red algal Rubisco from Griffithsia monillis in tobacco plant by fully replacement of native Rubisco large subunit gene in plastid genome with foreign Rubisco genes. Unfortunately, red algal Rubisco did not expressed as a functional form of L8S8. This result is the same as that Galdieria sulphuraria and Phaeodactylum tricornutum Rubiscos failed to assemble into a functional form in tobacco with coexpression of native tobacco Rubisco subunits genes, but this should be significant from standpoint of importance that native Rubisco large subunit gene was fully replaced with G. monillis Rubisco subunits genes.
This manuscript provides biological significant information to achieve functional expression of red algal Rubisco in plant plastid. The manuscript is well written and easy to follow. However, the authors need to consider the following comments and revise the manuscript.

Major:
1. I cannot understand a fate of large and small subunits of G. monilis Rubisco after translation. Author proposed that both subunits failed to assemble into functional L8S8 structure and were degraded. However, western blotting was performed using only soluble protein from leaf. Did both subunits that failed to assemble localized in insoluble fraction? In some case, unfolded or unassembled proteins are expressed as insoluble proteins.

Authors' response: We have now performed SDS-PAGE of insoluble protein fractions and added Figure 6. We did indeed observe the red algal large subunits in the insoluble fractions. We also observed a band consistent with CbbX only in the lines expressing its gene. We modified the "Blue-native PAGE and Immunoblot" part in the "Methods" section to "Blue-native PAGE, SDS-PAGE and Immunoblots" to include the additional

experiment. We also added one paragraph in the third part “The *Griffithsia monilis* Rubisco subunits failed to assemble into functional Rubisco in tobacco chloroplasts” of the “Results” section to describe the findings. We did not perform quantification of the large subunits observed in soluble versus insoluble fractions since we don’t have purified Rubisco from G. monilis available to use as a standard.

2. G. monilis large subunit of Rubisco was detected as smears at lower molecular weights in the western blot of Native PAGE in Fig. 5b. Especially, signal intensity of GmLN was much higher than those of other transformants. Author should mention this fact and explain a reason. I wonder if the modification of N-terminal of large subunit effected on translation efficiency. Authors described that a primary antibody used western blot recognizes Rubisco large subunits form multiple species including plants and cyanobacteria. Did author confirm that this antibody also recognizes Rubisco large subunits from red algae including G. monilis Rubisco? If this antibody was purchased, authors should mention a detail.

Authors’ response: We added the following in the second paragraph of part 3 of the “Results” section for possible explanation of stronger signal intensity of GmLN: “Interestingly, the signal from the GmL^{N} sample, which has the red algal large subunit with a modified N-terminus, is much stronger possibly due to improved translation efficiency or stability conferred by the modified N-terminus”.

The immunoblot of the SDS-PAGE in Figure 6B confirmed that the antibody was able to detect the Rubisco large subunit from *G. monilis*. We obtained the antibody from Professor Martin Parry’s lab and added the information in “Blue-native PAGE, SDS PAGE and Immunoblots” of the “Methods” section.

3. In Fig. 3, Gm-rbcS mRNAs were detected by RNA blot. I cannot understand why band sizes for Gm-rbcS mRNAs (shown as S in Fig. 3) were different between GmLS and GmLSX series. If my understanding is right, band sizes should be same. Author should explain for this.

Authors’ response: The difference in *Gm-rbcS* mRNAs band sizes between GmLS and GmLSX was due to the different terminators that follow *Gm-rbcS*. The *Nt-TrbcL* terminator leads to a larger *Gm-rbcS* transcript in GmLS compared to GmLSX series. The effect is also visible in *Gm-rbcL* transcripts (larger in GmL and GmL^{N}) although much less pronounced. We added “Slight variations in sizes among similar transcripts (e.g., *Gm-rbcS*) were due to their different terminators.” to the figure legend to clarify the issue.

Minor,
1. Description of genes such as "rbcL" and "rbcS", "rbc" should be italic through the whole text.

Authors’ response: We modified the gene description in italic accordingly.

2. A part of name of DNA restriction enzymes originated from organism name should be italic through the whole text.

Authors’ response: We modified the names of restriction enzymes such as *Nde*I, *Nhe*I and *Mau*BI accordingly.

3. Page 10, 2-carboxy-D-arabinitol 1-phosphate, "D" should be small capital.

Authors’ response: We modified “2-carboxy-D-arabinitol 1-phosphate” in paragraph 7 of the “Discussion” section accordingly.
